# Interaction of Hydrogen Sulfide with Oxygen Sensing under Hypoxia

**DOI:** 10.1155/2015/758678

**Published:** 2015-05-11

**Authors:** Bo Wu, Huajian Teng, Li Zhang, Hong Li, Jing Li, Lina Wang, Hongzhu Li

**Affiliations:** ^1^Department of Pathophysiology, Harbin Medical University, Harbin 150086, China; ^2^Joint Research Center for Bone and Joint Disease, Model Animal Research Center, Nanjing University, Nanjing 210093, China; ^3^Department of Geriatrics, The Second Affiliated Hospital of Harbin Medical University, Harbin 150086, China

## Abstract

Based on the discovery of endogenous H_2_S production, many in depth studies show this gasotransmitter with a variety of physiological and pathological functions. Three enzymes, cystathionine *β*-synthase (CBS), cystathionine *γ*-lyase (CSE), and 3-mercaptopyruvate sulfurtransferase (MST), are involved in enzymatic production of H_2_S. Emerging evidence has elucidated an important protective role of H_2_S in hypoxic conditions in many mammalian systems. However, the mechanisms by which H_2_S senses and responses to hypoxia are largely elusive. Hypoxia-inducible factors (HIFs) function as key regulators of oxygen sensing, activating target genes expression under hypoxia. Recent studies have shown that exogenous H_2_S regulates HIF action in different patterns. The activation of carotid bodies is a sensitive and prompt response to hypoxia, rapidly enhancing general O_2_ supply. H_2_S has been identified as an excitatory mediator of hypoxic sensing in the carotid bodies. This paper presents a brief review of the roles of these two pathways which contribute to hypoxic sensing of H_2_S.

## 1. Introduction

H_2_S had been known mostly due to its unpleasant smell and fierce toxicity before its physiological importance was studied [1]. Production of H_2_S in mammalian cells had been considered almost as a metabolic waste, until the physiological roles of this small molecular were elucidated in the central nervous system and the cardiovascular system nearly two decades ago [2, 3]. H_2_S is now identified as a well-known gasotransmitter which plays a critical role in both physiological regulation and pathophysiological processes of different mammalian tissues [4, 5]. Endogenous H_2_S levels have been reported in different mammalian systems, ranging from 50 to 160 *μ*M [6, 7]. The enzymatic production of endogenous H_2_S were identified, mostly composed of cystathionine *β*-synthase (CBS) [8–10], cystathionine *γ*-lyase (CSE) [11–13], and 3-mercaptopyruvate sulfurtransferase (MST) [14–16].

H_2_S and oxygen (O_2_) are mutually exclusive on the Earth. During the Permian period, the depletion of oxygen and accumulation of H_2_S in the oceans and the air caused great extinctions of more than 90 percent of life species [1, 17]. Physicochemical reactions in living cells require enough molecular oxygen O_2_ for essential metabolic processes. O_2_ serves as the terminal electron acceptor in the system of oxidative phosphorylation which produces high-energy phosphate bond in ATP [18, 19]. Insufficient oxygen supply to tissues, organs, or cells constitutes hypoxia. Hypoxia is evoked by several kinds of causes which all lead to oxygen-partial-pressure (PO_2_) in arterial blood less than 40 mm Hg [19, 20]. Several lines of evidence point to an important protective role of H_2_S in hypoxic conditions. However, the mechanisms by which H_2_S senses and responses to hypoxia are largely elusive. A master regulator of hypoxia in mammalian cells is hypoxia-inducible factor-1 (HIF-1), which activates the transcription of >100 target genes under hypoxic contexts [18, 21]. HIF-1 is involved in H_2_S-mediated angiogenesis under hypoxia [22, 23]. With the remarkable sensitivity and the prompt speed to response to hypoxia, the carotid bodies play a unique role in O_2_ sensing [24, 25]. It has been shown that H_2_S is involved in the regulation of sensory activity in the carotid bodies [26, 27]. In genetically distinct rat models, variations in endogenous H_2_S levels have been reported to be closely correlated with variations of carotid body O_2_ sensing in both hypoxia-induced pulmonary edema and essential hypertension [28]. In this paper, we review physiological responses of H_2_S to hypoxia and focus particularly on the regulating effects of H_2_S on HIF-1 action and the O_2_ sensing role of H_2_S in the carotid bodies.

## 2. H_**2**_S and HIF-1 under Hypoxia

### 2.1. Hypoxia and Hypoxia-Inducible Factor-1

HIF-1 was first identified as the protein responsible for the hypoxia-evoked transcription of erythropoietin [29, 30]. HIF-1 is a heterodimer consisting of the inducibly regulated HIF-1*α* subunits and the constitutively expressed HIF-1*β* subunits [31]. Since the expression of HIF-1*β* is abundant in cells, HIF-1*α* determines the protein level and the transcriptional activity of HIF-1 [21]. The HIF-1 heterodimer combines with the hypoxia-responsive element in target genes, thus enhancing target genes transcription [21].

**Figure 1:**
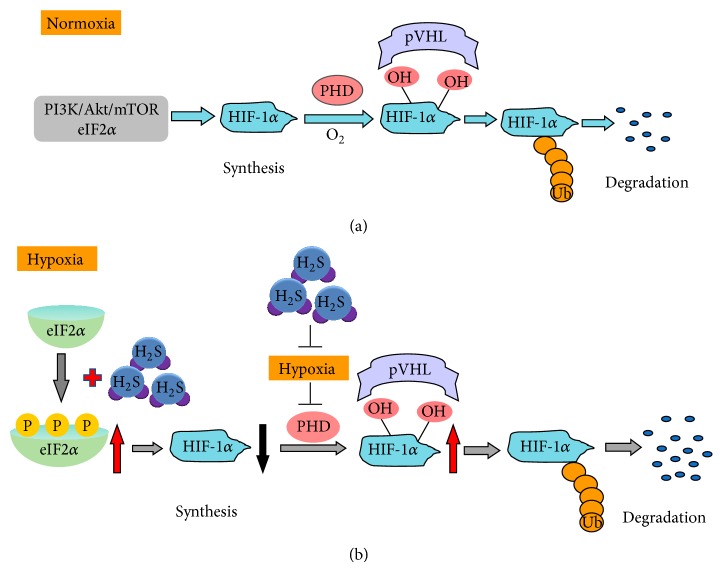
Pathways regulating HIF-1*α* synthesis/degradation under normoxia and schematic illustration of H_2_S effects on HIF-1*α* accumulation under hypoxia. (a) HIF-1*α* protein translation under normoxia is mainly dependent on activation of the PtdIns3K-Akt mammalian target of rapamycin (mTOR). HIF-1*α* is hydroxylated by the prolyl hydroxylase (PHD) under normoxia. Hydroxylated HIF-1*α* is then bound by the von Hippel Lindau protein (pVHL). This complex in turn recruits a ubiquitin ligase that targets HIF-1*α* for its proteasomal degradation. (b) Under hypoxia, H_2_S induces phosphorylation of translation initiation factor 2*α* (eIF2*α*). Phosphorylated eIF2*α* inhibits HIF-1*α* translation. In addition, H_2_S decreases cellular oxygen (O_2_) consumption under hypoxia and reverses hypoxia-induced inhibition of PHD activity. Thus, H_2_S enhances degradation of HIF-1*α*. Abbreviations: Ub, ubiquitin.

Under normoxic conditions, although continuously transcribed and translated, the protein levels of HIF-1*α* are hard to be detected in normal cells due to HIF-1*α* protein fast degradation. Tow specific proline residues within the oxygen-dependent degradation (ODD) domain in HIF-1*α* are hydroxylated by the prolyl hydroxylase (PHD) under normoxia. Hydroxylated HIF-1*α* is then bound by the von Hippel Lindau protein (VHL) [32]. This complex in turn recruits a ubiquitin ligase that targets HIF-1*α* for its proteasomal degradation [33] (Figure 1). PHD activity is inhibited under hypoxic conditions, thus resulting in HIF-1*α* stabilization. Other pathways and regulators are also involved in HIF-1*α* degradation [21].

Compared with the accumulated studies on HIF-1*α* degradation, translational control of stress-survival protein HIF-1*α* under hypoxia is far more beyond our understanding. HIF-1*α* protein translation under normoxia is dependent on activation of the PtdIns3 K-Akt mammalian target of rapamycin (mTOR) and the mitogen-activated protein kinases (MAPK) pathways [20, 21]. Under hypoxia-induced stress, phosphorylation of the translation initiation factor 2*α* (eIF2*α*) and inhibition of mTORC1 activity have been suggested to account for HIF-1*α* protein expression inhibition [21] (Figure 1). Certain antitumour compounds which inhibit HIF-1*α* translation contribute to eIF2*α* phosphorylation [34, 35]. There is evidence that other factors are involved in HIF-1*α* translation regulation, that is, studies on the 5′UTR of HIF-1*α* which contains an internal ribosome-entry-site [21].

### 2.2. Effects of H_2_S on HIF-1 Accumulation

Nitric oxide (NO), the first identified gasotransmitter, has been demonstrated to affect HIF-1 action [36, 37]. An early report showed that NO donors inhibited HIF-1 action under both hypoxia and hypoxia-mimetic conditions [36]. In later studies, by overexpressing the human iNOS to increase endogenous NO production, NO was shown to enhance HIF-1 protein accumulation and its activity [37]. The second gasotransmitter carbon monoxide (CO) has also been shown to regulate HIF-1 protein levels and activity in certain contexts [38]. A series of recent studies showed that H_2_S, like NO and CO, plays an important role in the regulation of HIF-1 function under hypoxia.* Caenorhabditis elegans *(*C. elegans*) is an attractive model for hypoxia relative research [6]. This animal model was used to study the influence of H_2_S on HIF-1. In* C. elegans*, the EGg laying defective- (EGL-) 9 is responsible for HIF-1 hydroxylation [39]. HIF-1 was closely correlated with increased tolerance of H_2_S exposure in* C. elegans* [39]. Animals with the HIF-1 null mutation did not survive from exposure to only 15 ppm H_2_S, while wild-type worms survived with high viability even exposure to 50 ppm H_2_S [39]. Mutations in either EGL-9 or VHL-1 induced an overabundance of HIF-1, which caused animals to tolerate 150 ppm H_2_S [39]. It is strongly indicated that HIF-1 is required when animals response to H_2_S. H_2_S treatment, as well as hypoxia, elevated the levels of HIF-1 protein and its activity [39]. However, H_2_S and hypoxia caused different expression patterns of a HIF-1 target gene [39].

The effects of H_2_S on HIF-1 in higher organisms have been investigated. In vascular smooth muscle cells, the interaction between H_2_S and HIF-1 was tested under CoCl_2_-induced hypoxia-mimetic conditions [40]. In a concentration of 300 *μ*M, NaHS induced upregulation of HIF-1*α* mRNA and proteins [40]. HIF-1*α* transcription activity in the same context was also increased [40]. The authors suggested that H_2_S upregulates HIF-1*α* gene transcription. However, HIF-1*α* synthesis during hypoxia is largely regulated at the level of translation rather than transcription [21]. It is worthy of mentioning that although CoCl_2_ treatment could induce HIF-1*α* accumulation to mimetic hypoxic stress, the model itself is not essential hypoxia and may cause other cellular changes [1].

Some later studies provided further understanding of the influence of H_2_S on HIF-1 in mammalian cells. It is reported that 1 mM NaHS (H_2_S donor) decreased hypoxia-induced HIF-1*α* protein accumulation and HIF-1 target gene expression in mammalian cell lines [22]. However, H_2_S had no similar effect on HIF-1 in hypoxia-mimetic conditions [22]. In contrast, another study showed that NaHS at relatively low concentrations (10–100 *μ*M) treatment decreased HIF-1*α* protein levels in HEK293T, Hep3B, and EA.hy926 cells under both hypoxia (1% O_2_) and hypoxia-mimetic conditions [23]. The controversial observations will be considered in subsequent sections.

### 2.3. Mechanisms of H_2_S Effects on HIF-1 Accumulation

In the* C. elegans* study, H_2_S-induced HIF-1 accumulation was dependent on EGL-9, indicating that H_2_S may prevent HIF-1 degradation [39]. In the same study, H_2_S-evoked HIF-1 increase was independent of VHL-1, which implies that* C. elegans* may have a special HIF-1 degradation pathway. However, hypoxia-mediated HIF-1 accumulation in mammals is largely dependent on VHL-1. It has been shown that VHL-independent degradation pathways play important roles in controlling HIF-1 levels [21]. CYSL-1 is homologous to CBS which is a key enzyme for H_2_S production in mammalian cells [41]. CYSL-1 identified from genetic screens negatively regulated EGL-9 and thus enhanced HIF-1 stability [41]. It was proposed that H_2_S promoted the interaction of EGL-9 and CYSL-1 to cause HIF-1 accumulation in* C. elegans* under hypoxia [41].

Kai and colleagues showed that H_2_S did not affect HIF-1 levels in EB8 cells under hypoxia, compared with control cells [22]. The fact that EB8 cells have no mtDNA suggested that mitochondria was involved in H_2_S-inhibited HIF-1 activation [22]. The authors found that 1 mM NaHS inhibited mitochondrial oxygen consumption and thus increased the oxygen level in hypoxic cells. H_2_S did not inhibit the stabilization of HIF-1*α* protein under hypoxia-mimetic conditions [22]. The authors concluded that H_2_S promoted HIF-1*α* degradation under hypoxia, due to NaHS-induced mitochondrial oxygen consumption inhibition (Figure 1). However, they did not provide the direct evidence that the speed of HIF-1*α* degradation was altered in the presence of H_2_S. On the other hand, it is worthy of mentioning that most of the key observations in their study were obtained with NaHS at 1 mM. This high concentration of H_2_S is clearly not within the physiological range of endogenous H_2_S and may have toxic effects on cells for long-time treatments [1, 42].

NaHS at 10–100 *μ*M has been used in different studies to reflect physiologically relevant concentrations of H_2_S in vivo [43, 44]. In a study using NaHS at 10–100 *μ*M, NaHS significantly lowered HIF-1*α* protein levels under both hypoxia (1% O_2_) and hypoxia-mimetic conditions (DFX or CoCl_2_) [23]. Although inducing HIF-1*α* accumulation, both DFX and CoCl_2_ have no effect on oxygen partial pressure in the culture medium [23]. It is suggested that oxygen partial pressure change or the alteration of mitochondrial respiration may not be involved in H_2_S-induced HIF-1*α* downregulation under hypoxia. Both HIF-1*α* degradation and HIF-1*α* ubiquitination were not changed by NaHS treatment [23]. In contrast, cycloheximide (CHX), a translation inhibitor, blocked the effect of NaHS on HIF-1*α* protein levels [23], suggesting that H_2_S mediates HIF-1*α* translation suppression. The authors further demonstrated that the key mechanism for H_2_S-induced HIF-1*α* downregulation was H_2_S-evoked repression of HIF-1*α* protein translation, rather than an effect on the ubiquitin proteasomal degradation pathway (Figure 1). Eukaryotic translation initiation factor 2*α* (eIF2*α*) is a critical regulatory molecule for eukaryotic initiation of translation [21]. eIF2*α* is responsible for the transformation of GDP to GTP, an essential step for translation start. Phosphorylation of eIF2*α* at Ser51 prevents the reformation of the eIF-2 ternary complex and thus inhibits protein translation [21]. Certain antitumour compounds which essentially decrease HIF-1*α* translation contribute to eIF2*α* phosphorylation [34, 35]. In eIF2*α* knockdown cells exposed to hypoxia, H_2_S-induced reduction of HIF-1*α* was partially reversed [23]. Therefore, HIF-1*α* translational suppression is associated with H_2_S-induced eIF2*α* phosphorylation in hypoxic contexts (Figure 1).

In summary of this section, it is likely that two key factors, the extent of hypoxia together with the concentration of H_2_S in the same condition, determine the final result of the action of H_2_S on HIF-1. H_2_S-induced upregulation of HIF-1 was observed in a* C. elegans* model.* C. elegans* naturally lives where O_2_ level is lower than that in the air [6, 7]. The animals have no circulatory system. O_2_ delivery in this species is dependent on diffusion.* C. elegans* are able to survive from O_2_ depletion, due to an anoxia-induced suspended animation state [6, 7]. In contrast, models in which H_2_S-triggered downregulation of HIF-1 come from high organisms which are highly oxygen-demanded. The differences in both species and oxygen demand may explain the opposite regulational patterns in the action of H_2_S on HIF-1. On the other hand, the effect of H_2_S on HIF-1 regulation may be dose-dependent. Differences in NaHS concentrations may be correlated with various observations in abovementioned studies. Thus, further studies on the role of endogenous H_2_S may provide further understanding of the interaction between H_2_S and HIF-1.

Emerging evidence indicates that VHL-independent HIF-1*α* degradation has an important role in controlling HIF-1*α* levels. Although this kind of pathways seems to be less dependent on oxygen levels in cells, that is, receptor of activated protein kinase C- (RACK1) mediated HIF-1*α* degradation [21], and further research is still required to solve whether these pathways are involved in the effect of H_2_S on HIF-1 in hypoxic contexts. On the other hand, translational regulation of HIF-1*α* under hypoxia is largely elusive. Therefore, whether H_2_S targets other factors which potentially regulate HIF-1*α* translation is another challenge. Moreover, it is not yet clear how H_2_S-mediated HIF-1*α* regulation contributes to the protective role of H_2_S under hypoxia.

## 3. H_**2**_S and Hypoxic Sensing in the Carotid Body

### 3.1. O_2_ Sensing in the Carotid Body

With the remarkable sensitivity and the fast speed to hypoxic response, the carotid body plays a unique role in O_2_ sensing [19]. Carotid bodies are small sensory organs located at the bifurcation of the common carotid artery [14, 19]. Changes in O_2_ levels of arterial blood rapidly active the carotid bodies, which in turn transduce sensory information to brainstem neurons [14]. The final response in the central nervous system regulates vital functions including breathing, heart rate, and blood pressure to increase ventilation and systemic delivery of oxygen [15]. Carotid bodies are primarily composed of two cell types: glomus cells (also called type I cells) and sustentacular cells (also called type II cells) [15]. Accumulated evidence suggests that type I cells are the primary site of hypoxic sensing in carotid bodies. NO and CO inhibited carotid body activity [15]. Neuronal nitric oxide synthase- (nNOS-) generated NO has been proposed as an important mediator of efferent inhibition of the carotid body [25, 45]. Heme oxygenase-2 (HO-2), a key enzyme for CO production, is expressed in glomus cells of many mammalian carotid bodies [24, 46]. Exogenous application of CO at low concentrations and HO inhibitors inhibited and stimulated the carotid body activity, respectively [16, 24].

### 3.2. Effects of CSE/H_2_S on Carotid Body Activity

**Figure 2:**
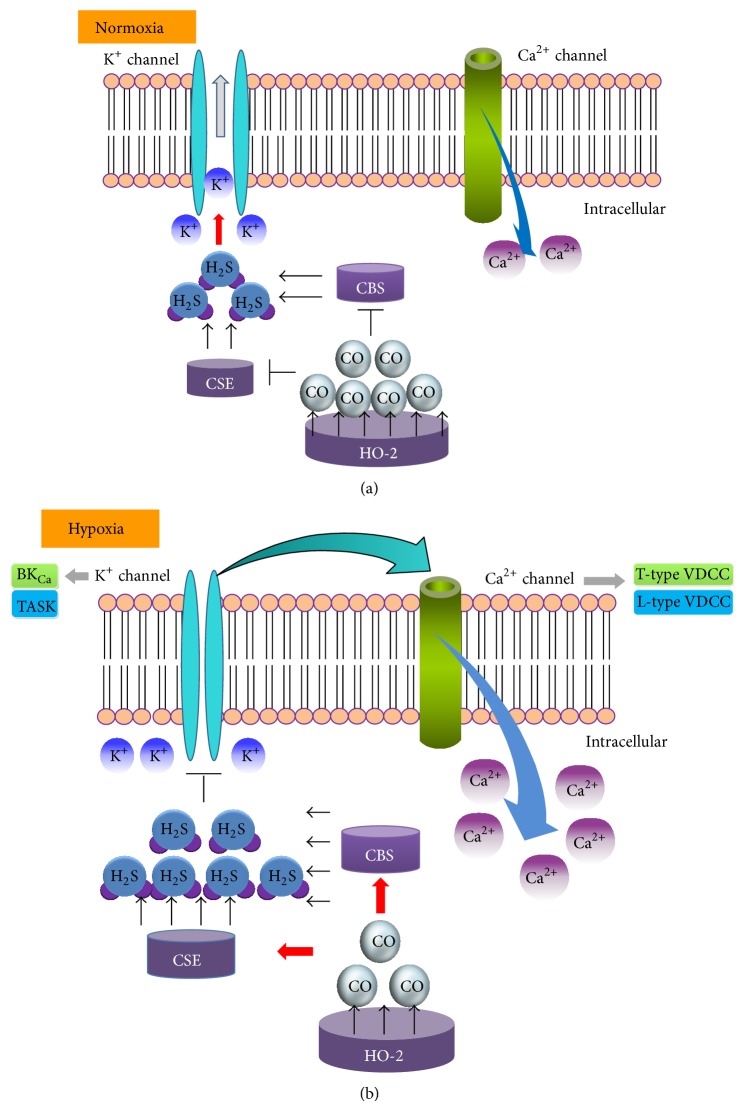
Potential interaction of heme oxygenase-2- (HO-2-) generated CO with cystathionine *γ*-lyase (CSE) and cystathionine *β*-synthase (CBS) in glomus cells of the carotid body. (a) Under normoxia, HO-2-generated CO is relatively high, resulting in inhibition of both CSE and CBS activity. H_2_S production is relatively low, contributing to low sensory activity in glomus cells. (b) Under hypoxia, CO generation from HO-2 is reduced, resulting in removal of CO inhibition on CSE and CBS activity. H_2_S levels are increased due to enhanced CSE and CBS activities. Increased H_2_S may activate Ca^2+^ channels due to membrane depolarization via the inhibition of K^+^ channels, which in turn excites sensory responses in glomus cells.

Emerging evidence demonstrated that H_2_S is involved in sensing and response to hypoxia in many tissues that possess the ability to sense hypoxia. H_2_S stimulated catecholamine secretion from chromaffin cells in trout [30]. H_2_S regulated the O_2_-sensing signal in trout chemoreceptors [47]. It was proposed that the balance between endogenous H_2_S production and its oxidation by available O_2_ contributed to H_2_S-mediated O_2_ sensing [47]. Recent studies showed that H_2_S, like NO and CO, is another gas regulator for hypoxic sensing in carotid bodies. Both CSE [26] and CBS proteins [27] are expressed in glomus cells. Rat carotid body expresses mRNAs for both CBS and CSE [48]. Hypoxia increased H_2_S generation in both mouse and rat carotid bodies [26]. Hypoxia-induced H_2_S production and secretion of catecholamine were significantly decreased in CSE knockout mice or in wide-type mice treated with CSE inhibitors DL-propargylglycine (PPG) [26]. CSE knockout mice exhibited severely impaired function of carotid body sensing to hypoxia. The similar observation was shown in rats treated with PPG [26]. Exogenous application of H_2_S donor, NaHS, increased the sensory excitation of the carotid bodies from mice and rats [26, 27]. The patterns of NaHS action on carotid bodies are similar to that of hypoxia. H_2_S produced by CSE may function as an excitatory mediator for the sensory excitation by hypoxia [26] (Figure 2).

### 3.3. Potential Effects of CBS/H_2_S on O_2_ Sensing in Carotid Body

It is worthy of mentioning that, in CSE knockout mice, the basal levels of H_2_S in the carotid bodies were half reduced compared to those of control wild-type animals [26]. It is likely that CBS may also be responsible for H_2_S production in the carotid bodies. Aminooxyacetic acid (AOA) and hydroxylamine (HA), two inhibitors of CBS, suppressed carotid body and ventilatory responses to hypoxia [27]. This study suggested that CBS-catalyzed H_2_S also plays a role in excitatory carotid bodies sensing under hypoxia (Figure 2). Compared to CSE inhibitor PPG, AOA and HA are nonspecific inhibitors [1]. HA inhibits many heme-containing enzymes [49, 50], while AOA generally targets aminotransferase [51, 52]. Given the pharmacologic nonspecific inhibition, further studies need to elucidate the effects of endogenous CBS on O_2_ sensing in carotid bodies. In addition, the relative importance of H_2_S/CSE and H_2_S/CBS on carotid bodies hypoxic sensing needs to be established.

The role of H_2_S/CBS in O_2_ sensing in tissues, other than carotid bodies, has been revealed in recent studies. CBS is a nuclear encoding heme protein [8]. The levels of CBS proteins in liver mitochondria are regulated in a Lon protease-mediated O_2_-dependent pathway [8]. Under normoxic conditions, a low level of CBS proteins was shown in mitochondria [8]. Hypoxia inhibited Lon protease activity and thus increased the accumulation of CBS in mitochondria [8]. Reoxygenation recovered Lon protease activity and thus accelerated the degradation of CBS proteins [8]. This study indicates that CBS accumulated in mitochondria is oxygen-sensitive. CBS serves as a CO-sensitive modulator of H_2_S in liver [53]. Studies using recombinant CBS indicated that CO bound to the prosthetic heme in CBS and thus specifically inhibited CBS activity [53]. In livers of heterozygous CBS knockout mice, overproducing CO had no effect on H_2_S generation. Another further study showed that CBS-catalyzed H_2_S functioned as a vasodilator in the cerebral circulation [54]. CO produced by HO-2 was O_2_-dependent in the brain. Endogenous CO negatively regulated CBS activity under normoxia, while, under hypoxia, CBS activity was increased due to the decrease of CO production [54]. However, it is not clear whether the interaction between CO and CBS affects the sensory action of carotid body.

### 3.4. Mechanisms of Regulating Carotid Body Activity by H_2_S

Recent studies showed that hypoxia induces the opening of voltage-dependent Ca^2+^ channels (VDCC) through inhibition of K^+^ channels in carotid bodies [14, 15]. VDCC activation increases intracellular Ca^2+^ concentration ([Ca^2+^]_i_) and thus enhances the release of neurotransmitters under hypoxia [15, 19] (Figure 2). NaHS inhibited big-conductance Ca^2+^-sensitive K^+^  (BK_Ca_) channels [27, 48], background K^+^ current (TASK) [55], and elevated [Ca^2+^]_i_ in glomus cells [55]. NaHS-induced elevation of [Ca^2+^]_i_ was abolished in the absence of extracellular Ca^2+^ [55, 56] or in case that the depolarization was prevented by voltage clamping at the resting membrane potential [55]. In cultured rat glomus cells, NaHS-induced elevation of [Ca^2+^]_i_ was blocked by nifedipine, a L-type VDCC blocker [56]. Similar finding was observed in both astrocytes [57] and rat neurons [58]. These findings support the view that L-type VDCC may be involved in H_2_S-induced elevation of [Ca^2+^]_i_ in carotid bodies [56] (Figure 2). A series of studies demonstrated that H_2_S was also involved in the activation of T-type VDCC [59–61]. More recently, it is suggested that Ca_V_3.2 T-type VDCC contributed to H_2_S mediated carotid body response to hypoxia [62]. Ca_V_3.2 was the major T-Type VDCC isoform expressed in the carotid body [62] (Figure 2). In brief, all abovementioned observations indicate that H_2_S may function as a physiological excitatory mediator of the carotid body sensory response to hypoxia (Figure 2). Moreover ion channels are closely correlated with this regulation (Figure 2).

In addition, exogenous application of H_2_S inhibited mitochondrial function in rat glomus cells [55]. The author proposed that the inhibitory effect of H_2_S on TASK was due to inhibition of oxidative phosphorylation. Given that mitochondrial reactive oxygen species is speculated to mediate carotid body action [63, 64], whether mitochondria is directly involved in H_2_S-mediated excitatory sensing in carotid bodies needs to be demonstrated. In addition, it is reported that neurotransmitters were involved in H_2_S-induced sensory excitation of carotid bodies. Acetylcholine (ACh) and adenosine triphosphate (ATP) are two excitatory neurotransmitters in the carotid bodies of both cats and rats [27, 65, 66]. Inhibition of purinergic receptors using pyridoxal phosphate-6-azophenyl-2′,4′-disulfonic acid, or application of hexamethonium, a blocker of nicotinic cholinergic receptors, prevented NaHS-evoked sensory excitation of the mouse carotid body [27]. This study indicates that the release of ATP/ACh from glomus cells is correlated with H_2_S-induced sensory excitation. However, Na_2_S (another H_2_S donors) significantly reduced the release of both ACh and ATP in the cat carotid body [65]. The effect of H_2_S on neurotransmitters release needs to be further established. Whether neurotransmitters release is associated with H_2_S-regulated carotid body activity is not clear.

### 3.5. Hypoxia-Induced H_2_S Increase: Evidence for Interaction of CSE with HO-2

In CSE knockout mice and rats treated with PPG, hypoxia failed to increase H_2_S generation in both mouse and rat carotid bodies [26]. These observations suggested that hypoxia-induced H_2_S production may be due to increased CSE activity. Hemeoxygenase-2 (HO-2) converts heme to CO [67]. HO-2 is constitutively expressed in type I cells of mammals [16]. It has been demonstrated that CO is an inhibitory mediator for carotid bodies hypoxia sensing [16] (Figure 2). Under normoxia, pharmacological inhibition of HO-2 greatly increased H_2_S generation [26]. In contrast, a CO donor inhibited the hypoxia-evoked H_2_S production in the carotid body [26]. These couple of findings suggested that CO may be a negative regulator for H_2_S production in the carotid body (Figure 2). In CSE knockout mice, the HO-2 inhibitor had no effect on H_2_S generation. Under hypoxia, reduced CO generation may improve H_2_S generation and the sensory action of the carotid body [26]. Taken together, it was proposed that H_2_S generation is negatively controlled by CO-inhibited CSE activity [26] (Figure 2). Given that CSE does not contain a heme group, this enzyme is unlikely to be bound by CO [1, 14]. To date, the mechanisms by which CO inhibits CSE activity are still unknown.

### 3.6. H_2_S-Mediated Sensory Activity of Carotid Bodies in Diseases

More recently, inherent variations in CO-mediated H_2_S have been shown to contribute to reflex variation of carotid body O_2_ sensing in three genetically distinct rat strains, including Sprague-Dawley (SD) rats, Brown-Norway (BN) rats, and spontaneous hypertensive (SH) rats [28]. In comparison with SD rats, BN rats display a profoundly reduced ventilatory response to hypoxia and also develop pulmonary edema when challenged with hypoxia [28, 68]. SH rats, compared with SD rats, exhibit an exaggerated response to hypoxia [69] and this alteration is vital for the development of essential hypertension [70]. Variability in kinetic properties of HO-2 was shown in liver microsomes from SD, BN, and SH rats, consistent with changes in CO levels from these three rat strains [28]. The authors proposed that variability of HO-2 activity of the liver was similar to that of the carotid bodies, based on the observation that hypoxic responses of the liver microsomes paralleled those of carotid bodies in the respective strain [28]. Impaired hypoxic sensitivity in BN rats was associated with augmented basal levels of CO and reduced H_2_S [28]. In contrast, the exaggerated hypoxic response in SH rats was correlated with lower CO and higher basal H_2_S [28]. Different substrate affinities for HO-2 resulted in various basal levels of CO and H_2_S generation [28]. Taken together, this series of findings further demonstrates that CO-H_2_S-mediated O_2_ sensing is a fundamental mechanism in the carotid body chemosensory reflex [28].

## 4. Summary and Perspective

An abundant and continuous supply of O_2_ is essential for survival of all mammalian cells. Reduced O_2_ availability or hypoxia evokes many important physiological responses and changes, for the sake of increasing oxygen delivery. The activation of carotid bodies is a sensitive and prompt response to hypoxia, rapidly enhancing general O_2_ supply. The family of HIFs, master regulators of cellular O_2_, induces an elegant series of delayed changes through activating target genes expression under hypoxia.

Emerging evidence shows that H_2_S plays a crucial role in O_2_ sensing, through regulating carotid body activity and HIF-1 action. The regulation of HIF-1 accumulation via exogenous H_2_S has been shown, but the mechanisms for this effect are largely elusive and controversial. The patterns for H_2_S-regulated HIF-1 may be dose-dependent. Further studies need to identify the role of endogenous H_2_S on HIF-1 action. Compared with many studies on the role of H_2_S on HIF-1*α*, few have been done to elucidate the interaction between H_2_S and HIF-2*α*. Glomus cells had higher expression of HIF-2*α* than that of HIF-1*α* [71]. Heterozygous HIF-1*α* gene-knockout mice had no carotid bodies responses to hypoxia [72]. Carotid bodies from mice with heterozygous knockout of HIF-2*α* gene exhibited exaggerated responses to hypoxia [73]. It was proposed that a balance between HIF-1*α* and HIF-2*α* contributed to changes of redox homeostasis in the carotid body [15]. Based on this hypothesis, would H_2_S affect HIF-1*α* and/or HIF-2*α* in the carotid body? Endogenously generated H_2_S has been shown as a stimulator of carotid body activity under hypoxia. Although CO has been shown to negatively regulate CSE-catalyzed H_2_S production, the accurate molecular mechanisms by which CO affects CSE activity are still unknown. Moreover, the effect of CBS/H_2_S on carotid body action is elusive.

One of the most recently identified mechanisms for H_2_S effect is *S*-sulfhydration [74–76]. The potential targets of *S*-sulfhydration in H_2_S-mediated O_2_ sensing remain to be determined. In addition, a novel study showed that H_2_S activated protein kinase G (PKG) I*α* oxidation by inducing disulfide formation [77]. The key mediator in that event is polysulfides. H_2_S rapidly converts to polysulfides when H_2_S contacts O_2_ or hydrogen peroxide [1, 77]. It is not yet clear whether polysulfides are involved in H_2_S-mediated carotid body response to hypoxia or H_2_S-regulated HIF functions.

## References

[1] Wang R. (2012). Physiological implications of hydrogen sulfide: a whiff exploration that blossomed. *Physiological Reviews*.

[2] Wang R. (2010). Hydrogen sulfide: the third gasotransmitter in biology and medicine. *Antioxidants and Redox Signaling*.

[3] Kimura H. (2010). Hydrogen sulfide: from brain to gut. *Antioxidants and Redox Signaling*.

[4] Olson K. R. (2014). Hydrogen sulfide as an oxygen sensor. *Clinical Chemistry and Laboratory Medicine*.

[5] Kimura H. (2014). Hydrogen sulfide and polysulfides as biological mediators. *Molecules*.

[6] Iranon N. N., Miller D. L. (2012). Interactions between oxygen homeostasis, food availability, and hydrogen sulfide signaling. *Frontiers in Genetics*.

[7] Shen C., Powell-Coffman J. A. (2003). Genetic analysis of hypoxia signaling and response in *C. elegans*. *Annals of the New York Academy of Sciences*.

[8] Teng H., Wu B., Zhao K., Yang G., Wu L., Wang R. (2013). Oxygen-sensitive mitochondrial accumulation of cystathionine *β*-synthase mediated by Lon protease. *Proceedings of the National Academy of Sciences of the United States of America*.

[9] Kimura Y., Kimura H. (2004). Hydrogen sulfide protects neurons from oxidative stress. *The FASEB Journal*.

[10] Wallace J. L. (2010). Physiological and pathophysiological roles of hydrogen sulfide in the gastrointestinal tract. *Antioxidants and Redox Signaling*.

[11] Yang G., Wu L., Bryan S., Khaper N., Mani S., Wang R. (2010). Cystathionine gamma-lyase deficiency and overproliferation of smooth muscle cells. *Cardiovascular Research*.

[12] Yang G., Cao K., Wu L., Wang R. (2004). Cystathionine gamma-lyase overexpression inhibits cell proliferation via a H_2_S-dependent modulation of ERK1/2 phosphorylation and p21 Cip/WAK-1. *The Journal of Biological Chemistry*.

[13] Yang G., Wu L., Jiang B. (2008). H_2_S as a physiologic vasorelaxant: hypertension in mice with deletion of cystathionine *γ*-lyase. *Science*.

[14] Semenza G. L., Prabhakar N. R. (2012). Gas biology: small molecular medicine. *Journal of Molecular Medicine*.

[15] Prabhakar N. R., Semenza G. L. (2012). Gaseous messengers in oxygen sensing. *Journal of Molecular Medicine*.

[16] Prabhakar N. R., Dinerman J. L., Agani F. H., Snyder S. H. (1995). Carbon monoxide: a role in carotid body chemoreception. *Proceedings of the National Academy of Sciences of the United States of America*.

[17] Olson K. R., Whitfield N. L. (2010). Hydrogen sulfide and oxygen sensing in the cardiovascular system. *Antioxidants and Redox Signaling*.

[18] Semenza G. L. (2011). Hypoxia. Cross talk between oxygen sensing and the cell cycle machinery. *The American Journal of Physiology—Cell Physiology*.

[19] Prabhakar N. R. (2013). Sensing hypoxia: Physiology, genetics and epigenetics. *Journal of Physiology*.

[20] Rocha S. (2007). Gene regulation under low oxygen: holding your breath for transcription. *Trends in Biochemical Sciences*.

[21] Koh M. Y., Spivak-Kroizman T. R., Powis G. (2008). HIF-1 regulation: not so easy come, easy go. *Trends in Biochemical Sciences*.

[22] Kai S., Tanaka T., Daijo H. (2012). Hydrogen sulfide inhibits hypoxia-but not anoxia-induced hypoxia-inducible factor 1 activation in a von Hippel-Lindau-and mitochondria-dependent manner. *Antioxidants and Redox Signaling*.

[23] Wu B., Teng H., Yang G., Wu L., Wang R. (2012). Hydrogen sulfide inhibits the translational expression of hypoxia-inducible factor-1alpha. *British Journal of Pharmacology*.

[24] Ortega-Sáenz P., Pascual A., Gómez-Díaz R., López-Barneo J. (2006). Acute oxygen sensing in heme oxygenase-2 null mice. *Journal of General Physiology*.

[25] Prabhakar N. R., Kumar G. K., Chang C. H., Agani F. H., Haxhiu M. A. (1993). Nitric oxide in the sensory function of the carotid body. *Brain Research*.

[26] Peng Y. J., Nanduri J., Raghuraman G. (2010). H_2_S mediates O_2_ sensing in the carotid body. *Proceedings of the National Academy of Sciences United States of America*.

[27] Li Q., Sun B., Wang X. (2010). A crucial role for hydrogen sulfide in oxygen sensing via modulating large conductance calcium-activated potassium channels. *Antioxidants and Redox Signaling*.

[28] Peng Y. J., Makarenko V. V., Nanduri J. (2014). Inherent variations in Co-H_2_S-mediated carotid body O_2_ sensing mediate hypertension and pulmonary edema. *Proceedings of the National Academy of Sciences of the United States of America*.

[29] Wang G. L., Semenza G. L. (1993). Desferrioxamine induces erythropoietin gene expression and hypoxia-inducible factor 1 DNA-binding activity: implications for models of hypoxia signal transduction. *Blood*.

[30] Perry S. F., McNeill B., Elia E., Nagpal A., Vulesevic B. (2009). Hydrogen sulfide stimulates catecholamine secretion in rainbow trout (*Oncorhynchus mykiss*). *The American Journal of Physiology—Regulatory Integrative and Comparative Physiology*.

[31] Wang G. L., Jiang B. H., Rue E. A., Semenza G. L. (1995). Hypoxia-inducible factor 1 is a basic-helix-loop-helix-PAS heterodimer regulated by cellular O_2_ tension. *Proceedings of the National Academy of Sciences of the United States of America*.

[32] Semenza G. L. (2006). Regulation of physiological responses to continuous and intermittent hypoxia by hypoxia-inducible factor 1. *Experimental Physiology*.

[33] Peers C., Wyatt C. N., Evans A. M. (2010). Mechanisms for acute oxygen sensing in the carotid body. *Respiratory Physiology and Neurobiology*.

[34] Jung H. J., Park J. W., Lee J. S. (2009). Silibinin inhibits expression of HIF-1alpha through suppression of protein translation in prostate cancer cells. *Biochemical and Biophysical Research Communications*.

[35] Zhang J., Cao J., Weng Q. (2010). Suppression of hypoxia-inducible factor 1alpha (HIF-1alpha) by tirapazamine is dependent on eiF2alpha phosphorylation rather than the mTORC1/4E-BP1 pathway. *PLoS ONE*.

[36] Hagen T., Taylor C. T., Lam F., Moncada S. (2003). Redistribution of intracellular oxygen in hypoxia by nitric oxide: effect on HIF1alpha. *Science*.

[37] Metzen E., Zhou J., Jelkmann W., Fandrey J., Brüne B. (2003). Nitric oxide impairs normoxic degradation of HIF-1alpha by inhibition of prolyl hydroxylases. *Molecular Biology of the Cell*.

[38] Choi Y. K., Kim C.-K., Lee H. (2010). Carbon monoxide promotes VEGF expression by increasing HIF-1alpha protein level via two distinct mechanisms, translational activation and stabilization of HIF-1alpha protein. *The Journal of Biological Chemistry*.

[39] Budde M. W., Roth M. B. (2010). Hydrogen sulfide increases hypoxia-inducible factor-1 activity independently of von Hippel-Lindau tumor suppressor-1 in *C. elegans*. *Molecular Biology of the Cell*.

[40] Liu X. H., Pan L. L., Zhuo Y., Gong Q. H., Rose P., Zhu Y. Z. (2010). Hypoxia-inducible factor-1alpha is involved in the pro-angiogenic effect of hydrogen sulfide under hypoxic stress. *Biological and Pharmaceutical Bulletin*.

[41] Ma D. K., Vozdek R., Bhatla N., Horvitz H. R. (2012). CYSL-1 interacts with the O_2_-sensing hydroxylase EGL-9 to promote H_2_S-modulated hypoxia-induced behavioral plasticity in *C. elegans*. *Neuron*.

[42] Moore P. K., Bhatia M., Moochhala S. (2003). Hydrogen sulfide: from the smell of the past to the mediator of the future?. *Trends in Pharmacological Sciences*.

[43] Yang G., Li H., Tang G. (2012). Increased neointimal formation in cystathionine gamma-lyase deficient mice: role of hydrogen sulfide in alpha5beta1-integrin and matrix metalloproteinase-2 expression in smooth muscle cells. *Journal of Molecular and Cellular Cardiology*.

[44] Yang G., Yang W., Wu L., Wang R. (2007). H_2_S, endoplasmic reticulum stress, and apoptosis of insulin-secreting beta cells. *The Journal of Biological Chemistry*.

[45] Campanucci V. A., Nurse C. A. (2007). Autonomic innervation of the carotid body: Role in efferent inhibition. *Respiratory Physiology and Neurobiology*.

[46] Mkrtchian S., Kåhlin J., Ebberyd A. (2012). The human carotid body transcriptome with focus on oxygen sensing and inflammation—a comparative analysis. *Journal of Physiology*.

[47] Olson K. R., Dombkowski R. A., Russell M. J. (2006). Hydrogen sulfide as an oxygen sensor/transducer in vertebrate hypoxic vasoconstriction and hypoxic vasodilation. *Journal of Experimental Biology*.

[48] Telezhkin V., Brazier S. P., Cayzac S. H., Wilkinson W. J., Riccardi D., Kemp P. J. (2010). Mechanism of inhibition by hydrogen sulfide of native and recombinant BKCa channels. *Respiratory Physiology and Neurobiology*.

[49] Prabhakar N. R. (2012). Hydrogen sulfide (H_2_S): a physiologic mediator of carotid body response to hypoxia. *Advances in Experimental Medicine and Biology*.

[50] Donzelli S., Espey M. G., Flores-Santana W. (2008). Generation of nitroxyl by heme protein-mediated peroxidation of hydroxylamine but not N-hydroxy-L-arginine. *Free Radical Biology and Medicine*.

[51] Schwarcz R., Eid T., Du F. (2000). Neurons in layer III of the entorhinal cortex. A role in epileptogenesis and epilepsy?. *Annals of the New York Academy of Sciences*.

[52] McKenna M. C., Waagepetersen H. S., Schousboe A., Sonnewald U. (2006). Neuronal and astrocytic shuttle mechanisms for cytosolic-mitochondrial transfer of reducing equivalents: Current evidence and pharmacological tools. *Biochemical Pharmacology*.

[53] Shintani T., Iwabuchi T., Soga T. (2009). Cystathionine beta-synthase as a carbon monoxide-sensitive regulator of bile excretion. *Hepatology*.

[54] Morikawa T., Kajimura M., Nakamura T. (2012). Hypoxic regulation of the cerebral microcirculation is mediated by a carbon monoxide-sensitive hydrogen sulfide pathway. *Proceedings of the National Academy of Sciences of the United States of America*.

[55] Buckler K. J. (2012). Effects of exogenous hydrogen sulphide on calcium signalling, background (TASK) K channel activity and mitochondrial function in chemoreceptor cells. *Pflugers Archiv European Journal of Physiology*.

[56] Makarenko V. V., Nanduri J., Raghuraman G. (2012). Endogenous H_2_S is required for hypoxic sensing by carotid body glomus cells. *The American Journal of Physiology—Cell Physiology*.

[57] Nagai Y., Tsugane M., Oka J.-I., Kimura H. (2004). Hydrogen sulfide induces calcium waves in astrocytes. *The FASEB Journal*.

[58] García-Bereguiaín M. A., Samhan-Arias A. K., Martín-Romero F. J., Gutiérrez-Merino C. (2008). Hydrogen sulfide raises cytosolic calcium in neurons through activation of L-type Ca^2+^ channels. *Antioxidants and Redox Signaling*.

[59] Kawabata A., Ishiki T., Nagasawa K. (2007). Hydrogen sulfide as a novel nociceptive messenger. *Pain*.

[60] Matsunami M., Tarui T., Mitani K. (2009). Luminal hydrogen sulfide plays a pronociceptive role in mouse colon. *Gut*.

[61] Maeda Y., Aoki Y., Sekiguchi F. (2009). Hyperalgesia induced by spinal and peripheral hydrogen sulfide: evidence for involvement of Cav3.2 T-type calcium channels. *Pain*.

[62] Makarenko V. V., Peng Y. J., Yuan G. (2015). CaV3.2 T-type Ca^2+^ channels in H_2_S-mediated hypoxic response of the carotid body. *The American Journal of Physiology—Cell Physiology*.

[63] He L., Dinger B., Gonzalez C., Obeso A., Fidone S. (2006). Function of NADPH oxidase and signaling by reactive oxygen species in rat carotid body type I cells. *The Arterial Chemoreceptors*.

[64] Prabhakar N. R. (2011). Sensory plasticity of the carotid body: role of reactive oxygen species and physiological significance. *Respiratory Physiology & Neurobiology*.

[65] Fitzgerald R. S., Shirahata M., Chang I., Kostuk E. (2009). The impact of hypoxia and low glucose on the release of acetylcholine and ATP from the incubated cat carotid body. *Brain Research*.

[66] Alcayaga C., Varas R., Valdés V. (2007). ATP- and ACh-induced responses in isolated cat petrosal ganglion neurons. *Brain Research*.

[67] Williams S. E. J., Wootton P., Mason H. S. (2004). Hemoxygenase-2 is an oxygen sensor for a calcium-sensitive potassium channel. *Science*.

[68] Strohl K. P., Thomas A. J., St. Jean P., Schlenker E. H., Koletsky R. J., Schork N. J. (1997). Ventilation and metabolism among rat strains. *Journal of Applied Physiology*.

[69] Hayward L. F., Castellanos M., Noah C. (2012). Cardiorespiratory variability following repeat acute hypoxia in the conscious SHR versus two normotensive rat strains. *Autonomic Neuroscience: Basic and Clinical*.

[70] Tan Z. Y., Lu Y., Whiteis C. A. (2010). Chemoreceptor hypersensitivity, sympathetic excitation, and overexpression of ASIC and TASK channels before the onset of hypertension in SHR. *Circulation Research*.

[71] Kumar P., Prabhakar N. R. (2012). Peripheral chemoreceptors: function and plasticity of the carotid body. *Comprehensive Physiology*.

[72] Kline D. D., Peng Y.-J., Manalo D. J., Semenza G. L., Prabhakar N. R. (2002). Defective carotid body function and impaired ventilatory responses to chronic hypoxia in mice partially deficient for hypoxia-inducible factor 1alpha. *Proceedings of the National Academy of Sciences of the United States of America*.

[73] Peng Y. J., Nanduri J., Khan S. A. (2011). Hypoxia-inducible factor 2alpha (HIF-2alpha) heterozygous-null mice exhibit exaggerated carotid body sensitivity to hypoxia, breathing instability, and hypertension. *Proceedings of the National Academy of Sciences of the United States of America*.

[74] Altaany Z., Ju Y., Yang G., Wang R. (2014). The coordination of S-sulfhydration, S-nitrosylation, and phosphorylation of endothelial nitric oxide synthase by hydrogen sulfide. *Science Signaling*.

[75] Zhao K., Ju Y., Li S., Altaany Z., Wang R., Yang G. (2014). S-sulfhydration of MEK1 leads to PARP-1 activation and DNA damage repair. *EMBO Reports*.

[76] Gadalla M. M., Snyder S. H. (2010). Hydrogen sulfide as a gasotransmitter. *Journal of Neurochemistry*.

[77] Stubbert D., Prysyazhna O., Rudyk O., Scotcher J., Burgoyne J. R., Eaton P. (2014). Protein kinase G I*α* oxidation paradoxically underlies blood pressure lowering by the reductant hydrogen sulfide. *Hypertension*.

